# Endocannabinoids Measurement in Human Saliva as Potential Biomarker of Obesity

**DOI:** 10.1371/journal.pone.0042399

**Published:** 2012-07-31

**Authors:** Isabelle Matias, Blandine Gatta-Cherifi, Antoine Tabarin, Samantha Clark, Thierry Leste-Lasserre, Giovanni Marsicano, Pier Vincenzo Piazza, Daniela Cota

**Affiliations:** 1 Group “Energy Balance and Obesity”, Institut National de la Santé et de la Recherche Médicale (INSERM), Neurocentre Magendie, Physiophatologie de la Plasticité Neuronale, Bordeaux, France; 2 Group “Endocannabinoids and Neuroadaptation”, Institut National de la Santé et de la Recherche Médicale (INSERM), Neurocentre Magendie, Physiophatologie de la Plasticité Neuronale, Bordeaux, France; 3 Group “Physiopathology of Addiction”, Institut National de la Santé et de la Recherche Médicale (INSERM), Neurocentre Magendie, Physiophatologie de la Plasticité Neuronale, Bordeaux, France; 4 University of Bordeaux, Neurocentre Magendie, Physiopathologie de la Plasticité Neuronale, Bordeaux, France; 5 Endocrinology Department, Haut-Lévêque Hospital, Pessac, France; Sapienza University of Rome, Italy

## Abstract

**Background:**

The discovery of the endocannabinoid system and of its role in the regulation of energy balance has significantly advanced our understanding of the physiopathological mechanisms leading to obesity and type 2 diabetes. New knowledge on the role of this system in humans has been acquired by measuring blood endocannabinoids. Here we explored endocannabinoids and related *N*-acylethanolamines in saliva and verified their changes in relation to body weight status and in response to a meal or to body weight loss.

**Methodology/Principal Findings:**

Fasting plasma and salivary endocannabinoids and *N*-acylethanolamines were measured through liquid mass spectrometry in 12 normal weight and 12 obese, insulin-resistant subjects. Salivary endocannabinoids and *N*-acylethanolamines were evaluated in the same cohort before and after the consumption of a meal. Changes in salivary endocannabinoids and *N*-acylethanolamines after body weight loss were investigated in a second group of 12 obese subjects following a 12-weeks lifestyle intervention program. The levels of mRNAs coding for enzymes regulating the metabolism of endocannabinoids, *N*-acylethanolamines and of cannabinoid type 1 (CB_1_) receptor, alongside endocannabinoids and *N*-acylethanolamines content, were assessed in human salivary glands.

The endocannabinoids 2-arachidonoylglycerol (2-AG), *N-*arachidonoylethanolamide (anandamide, AEA), and the *N*-acylethanolamines (oleoylethanolamide, OEA and palmitoylethanolamide, PEA) were quantifiable in saliva and their levels were significantly higher in obese than in normal weight subjects. Fasting salivary AEA and OEA directly correlated with BMI, waist circumference and fasting insulin. Salivary endocannabinoids and *N*-acylethanolamines did not change in response to a meal. CB_1_ receptors, ligands and enzymes were expressed in the salivary glands. Finally, a body weight loss of 5.3% obtained after a 12-weeks lifestyle program significantly decreased salivary AEA levels.

**Conclusions/Significance:**

Endocannabinoids and *N*-acylethanolamines are quantifiable in saliva and their levels correlate with obesity but not with feeding status. Body weight loss significantly decreases salivary AEA, which might represent a useful biomarker in obesity.

## Introduction

The discovery of the endocannabinoid system (ECS) and of its impact on the regulation of energy homeostasis represents a significant advance in the study of obesity and type 2 diabetes [1]–[4].

The ECS comprises two distinct membrane cannabinoid receptors, CB_1_ and CB_2_, specific ligands named endocannabinoids, such as anandamide (AEA) and 2-arachidonoylglycerol (2-AG), and enzymes for ligand biosynthesis and inactivation [5]. Endocannabinoids have known appetite-stimulating effects in animals by acting at cannabinoid CB_1_ receptors [1], [2]. Furthermore, there is evidence for an up-regulation of endocannabinoids and endocannabinoid-related N-acylethanolamines oleoylethanolamide (OEA) and palmitoylethanolamide (PEA) in both blood and adipose tissue of obese humans [6]–[9]. Recently, we have shown that changes in plasma AEA levels might have physiological relevance in the anticipatory or preparatory phase of the meal in humans, since both normal weight and obese subjects have a pre-prandial peak in plasma AEA levels [10]. However, obese subjects show blunted post-prandial plasma AEA and peptide-YY (PYY) changes, suggesting that the concomitant deregulation of both orexigenic and anorexigenic systems contributes to the establishment of the obese phenotype [10].

The saliva is the first digestive secretion produced in response to the ingestion of food [11]. Therefore, it is reasonable to investigate whether signals and systems involved in the regulation of food intake, such as the ECS, might be present in saliva and exert a functional role. Besides, saliva offers distinctive advantages over serum or plasma as a diagnostic tool, thanks to the non-invasiveness of the collection procedure.

The saliva is produced and secreted by the three pairs of major salivary glands (parotid, submandibular, sublingual) and minor salivary glands after the stimulation of both sympathetic and parasympathetic nervous system to protect the mouth and to participate to the digestion [11], [12]. It is composed mostly of water, but it also contains important compounds such as electrolytes, mucus, various enzymes for food tasting and digestion, bacteria for mucosa protection and serum derivatives, including hormones [11], [12]. The saliva therefore derives from both local and systemic sources and is often collected for the diagnosis of systemic pathologies [11], [12].

Interestingly, gastrointestinal hormones known to play key roles in food intake and energy balance, like PYY and ghrelin, have been identified in human saliva [13], [14]. Analysis of salivary ghrelin levels in obese subjects undergoing body weight loss through bariatric surgery has demonstrated that there is an autonomous production of ghrelin in salivary glands, which is irrespective of nutritional status and weight loss [15]. While, salivary PYY is synthesized in the taste cells of the mouse tongue and its Y2R receptor is expressed in von Ebner's lingual salivary glands [13]. Interestingly, gene-transfer mediated increase in mouse salivary PYY production significantly decreases food intake [13].

At present, there is no information concerning the presence and potential role of the ECS in human saliva and salivary glands. This is possibly due to the lack of a sensitive analytical method that has not allowed the reliable quantification of endocannabinoids and related compounds in human saliva [16], [17]. Nonetheless, endocannabinoids have been detected in the salivary glands of the lone star tick [18] and the rat [19] and CB_1_ receptors have been localized in the major salivary glands of both rats [20] and dogs [21]. In addition, there is some evidence suggesting that the ECS might inhibit salivary secretion [20], [22]. In this respect, one of the major side effects of marijuana intoxication in humans is a decrease of saliva production (dry mouth) [23].

In the present study we therefore sought to determine whether quantification of endocannabinoids and *N*-acylethanolamines in saliva is feasible and reliable, and whether salivary endocannabinoids and *N*-acylethanolamines levels might differ between normal weight and obese subjects and might change in relation to the consumption of a meal or to body weight loss.

## Methods

### Subjects

The study was approved by the Haut-Lévêque Hospital Research Ethics Committee and all participants provided written informed consent.

The characteristics of the 24 subjects (12 normal weight and 12 non diabetic, insulin-resistant obese) participating to the meal study were as previously described [10]. The meal was designed by a hospital nutritionist to guarantee that, independently from the food items consumed, it contained an equal amount of macronutrients (35% lipids, 45% carbohydrates and 20% proteins) and calories. The meal consisted of a starter (a small portion of salad or vegetables), a main dish (roasted pork, lamb, chicken, salmon or tuna) accompanied by pasta or rice and vegetables, white bread and a cheese and fruit portion. Subjects randomly consumed meals containing different food items, so to avoid any potential effect on endocannabinoids precursors' pools [24].

The lifestyle intervention program of the Haut-Lévêque Hospital, which is based on [25], [26], consists of 12 weekly sessions of group meetings with a dietician, a physical activity teacher, a psychologist and a medical doctor. The characteristics of the 12 obese subjects enrolled in the hospital lifestyle intervention program and whose salivary samples were used for the study are reported in Table 1. These 12 subjects included 4 patients with type 2 diabetes and 1 patient with fasting hyperglycemia. Eight of these patients were suffering from hypertension, while 5 had sleep apnea syndrome. Anthropometric measurements (weight, height, waist circumference) and blood pressure as well as blood samples for the analysis of biochemical parameters (blood glucose, lipid profile, liver enzymes) were collected at the beginning and at the end of the 12 weeks of the lifestyle intervention. Fasting salivary samples were also collected at the beginning and at the end of the 12 weeks. The subjects included in the study achieved a mean body weight loss of 5.33±0.97% at the end of the program.

**Table 1:** Characteristics of the subjects of the lifestyle intervention study.

	Obese before lifestyle intervention	Obese after lifestyle intervention
Sex (M/F)	4/8	4/8
Age (years)	45.0±2.5	45.0±2.5
Weight (Kg)	121.74±8.93	115.0±7.98**
BMI (Kg/m^2^)	44.3±3.1	41.8±2.8**
Waist circumference (cm)	123.5±5.3	118.7±5.2**
SBP (<140 mmHg)	127.3±7.5	118.3±3.9*
DBP (<90 mmHg)	74.7±3.9	71.1±2.5
Glucose (4.0–5.6 mmol/l)	5.97±0.21	5.6±0.20*
HDL cholesterol (1.0–1.80 mmol/l)	1.17±0.07	1.08±0.32
Triglycerides (0.3–1.7 mmol/l)	1.45±0.44	0.95±0.08*
ASAT (10–35 U/l)	20.25±1.67	16.25±1.19*
ALAT (5–40 U/l)	31.08±4.31	25.33±3.56**
Gamma-GT (5–38 UI/l)	33.42±6.58	25.55±4.33*

Values in parenthesis indicate normal range. SBP: systolic blood pressure; DBP: diastolic blood pressure; ASAT: aspartate aminotransferase; ALAT: alanine aminotransferase; gamma-GT: gamma-glutamyl transferase.

* *p*<0.05,

** *p*<0.01 before *vs*. after lifestyle intervention program.

Submandibular gland fragments and accessory salivary glands were obtained from 2 patients who respectively underwent maxillofacial surgery for suspicion of tumor and accessory salivary gland biopsy for suspicion of amyloidosis. No tissue collection was superimposed and leftover tissues were used. Only macroscopically intact tissues were used for subsequent PCR, endocannabinoids and *N*-acylethanolamines content analyses.

### Blood and saliva sampling

For the meal study, subjects underwent blood and saliva sampling after an overnight fast, soon after the arrival at the hospital (09:00 h). Saliva samples were then also collected 1 h before the meal (11:00 h), immediately before the meal (12:00 h) and 1 h after the termination of the meal. For the lifestyle intervention study, salivary samples were collected before and after body weight loss and always after an overnight fast. Subjects were asked to wash their mouth with water and to not drink or eat anything at least half hour before spitting into a falcon 15-ml tube. In all the studies, subjects were forbidden to tooth brush on the day of the salivary sample collection, so to avoid blood contamination of the samples. The salivary samples were immediately placed at 4°C without centrifugation and then kept at −80°C until analysis. Blood samples were collected in heparin tubes, immediately centrifuged at 4°C at 1500 g for 20 min and then kept at −80°C until analysis.

### Plasma lipids, glucose and liver enzymes measurements

Lipids, glucose and liver enzymes were determined by enzymatic and fluorometric methods, as described in [10].

### Endocannabinoids and N-acylethanolamines measurement in plasma, saliva and salivary glands

Endocannabinoids and *N*-acylethanolamines were extracted, purified and quantified following a set of different biochemical steps [27]. Briefly, human plasma, saliva or submandibular salivary glands were homogenized and extracted with chloroform/methanol (2∶1, v/v) containing internal deuterated standards (Cayman Chemicals, Ann Arbor, MI, USA) and then pre-purified by open bed chromatography if necessary. Tandem mass spectral analyses were performed on a TSQ Quantum Access triple quadrupole instrument (Thermo-Scientific, Waltham, MA, USA) equipped with an atmospheric pressure chemical ionization source and operating in positive ion mode. A new sensitive and specific liquid chromatography-tandem mass spectrometric analysis (LC-MS/MS) method was developed and validated [10]. Endocannabinoids and *N*-acylethanolamines were then quantified by isotopic dilution using a seven-point calibration curve and measurements expressed as pmol/ml of plasma or saliva, or fmol/mg or pmol/mg of tissue of salivary glands.

### RNA extraction and PCR procedure

RNA was extracted with Tri Reagent following the manufacturer's instructions (Fermentas, Villebon sur Yvette, France). After extraction and ethanol precipitation, RNA was treated with Turbo DNA-free (Ambion, Austin, TX, USA). The integrity of the RNA was checked by capillary electrophoresis using the RNA 6000 Nano Lab-on-a-Chip kit and the Bioanalyzer 2100 (Agilent Technologies, Palo Alto, CA, USA). cDNA was synthesized from 2 µg total RNA, with RevertAidTM Premium Reverse Transcriptase (Fermentas), using random hexamer primers and Oligo(dT)18 primer (Fermentas). The RT-PCR amplification of primers was carried out with a denaturation step at 95°C for 15 min, followed by 40 cycles of denaturation at 95°C for 30 s, primer annealing at 60°C (or 55°C depending on primer condition) for 30 s, and primer extension at 72°C for 1 min. Upon completion of the cycling steps, a final extension was done at 72°C for 5 min. RT-PCR was carried out using a PTC-200 Biorad Thermal Cycler. The primer (Eurogentec, Seraing, Belgium) sequences were the following: CB_1_, Forward (F): 5′ GAT GTC TTT GGG AAG ATG AAC AAG C 3′, Reverse (R): 5′ AGA CGT GTC TGT GGA CAC AGA CAT GG 3′; fatty acid amide hydrolase (FAAH), F: 5′ GTG GTG CT(G/A) ACC CCC ATG CTG G 3′, R: 5′ TCC ACC TCC CGC ATG AAC CGC AGA CA 3′; monoacylglycerol lipase (MAGL), F: 5′ TCT TCC TTC TGG GCC ACT CCA 3′, R: 5′ GGA TTG GCA AGA ACC AGA GG 3′; diacylglycerol lipase alpha (DAGL alpha) F: 5′ GCC ATC TTC CTC TTT CTC CTG 3′, R: 5′ GAG CAC GTA CTG CAT GGA GTC 3′; diacylglycerol lipase beta (DAGL beta) F: 5′ AAG CTG CCA GAT ACG TTT ACC 3′, R: 5′ GGA GAA GGC GTA GCA CCT GAC 3′; N-acyl phosphatidylethanolamine phospholipase D (NAPE-PLD) F: 5′ TCA AAC AGT AGA ACA GTG TGT ACG 3′, R: 5′ TTG AAT GAA AAA TGC TTA TGT ATT G 3′; N-acylethanolamine-hydrolyzing acid amidase (NAAA) F: 5′ AGC AGA CAT TTG GCC TCT AG 3′, R: TTC TCC GGT CAT CTT CCT T 3′; glyceraldehyde-phosphate dehydrogenase (GAPDH), F: 5′ CCC TTC ATT GAC CTC AAC TAC ATG GT 3′, R: 5′ GAG GGG CCA TCC ACA GTC TTC TG 3′; hypoxanthine phosphoribosyl transferase (HPRT), F: 5′ CTT GCT CGA GAT GT(C/G) ATG AAG 3′, R: 5′ TGC ATT GTT TT(A/G) CCA GTG 3′. Housekeeping genes GAPDH and HPRT were used as internal controls. PCR products were analyzed by electrophoresis on a 1.8% agarose gel.

### Statistical analysis

Statistical analysis was performed using Statistica version 8.0 (StatSoft, Tulsa, OK, USA). All values are reported as means ± SEM. For the meal study, data were analyzed by 1-way and 2-way repeated measurements ANOVA or by two-tailed Student's *t*-tests when appropriate. For the lifestyle intervention study, data were analyzed with paired t-test or wilcoxon test if data were not normally distributed. Significant ANOVAs were followed by Tukey post-hoc test. For correlation analysis, Pearson's or Spearman's were used when appropriate. *p* values less than 0.05 denote statistical significance.

## Results

### Endocannabinoids and N-acylethanolamines can be detected in saliva

**Figure 1:**
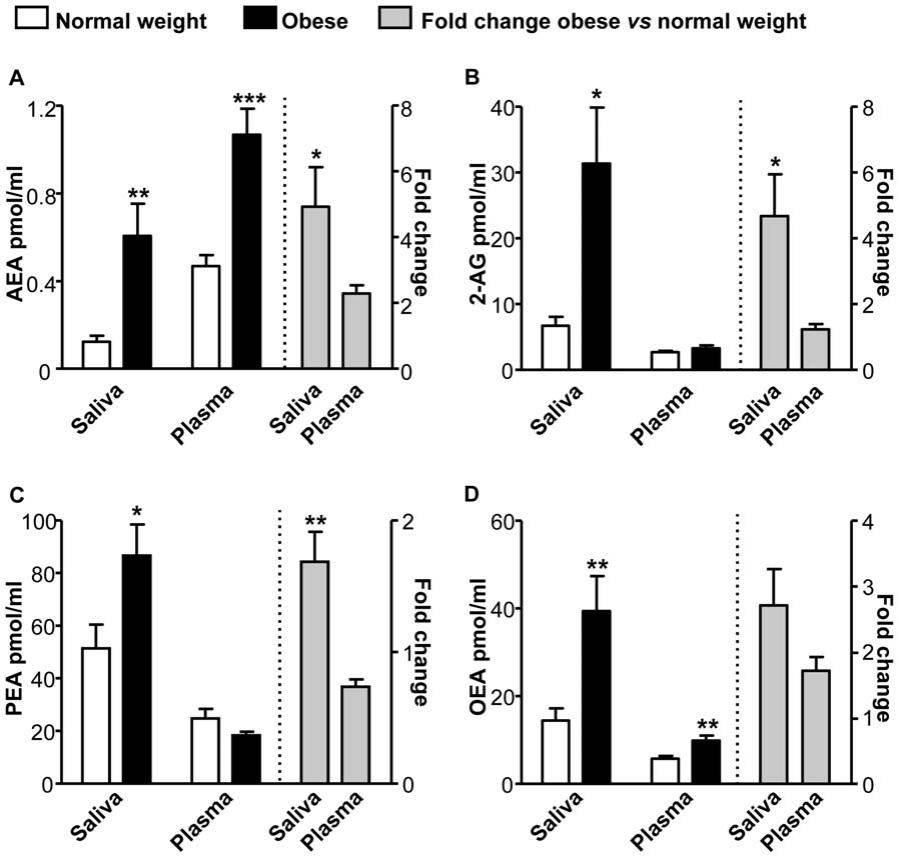
Fasting salivary endocannabinoids and *N*-acylethanolamines are elevated in obese subjects. Fasting salivary and plasma AEA (**A**), 2-AG (**B**), PEA (**C**) and OEA (**D**) levels in normal weight and obese subjects. *p<0.05, **p<0.01, ***p<0.001.

Salivary endocannabinoids and *N*-acylethanolamines were reliably detectable and quantifiable in human saliva (Figure 1). Fasting saliva endocannabinoids (AEA and 2-AG) and *N*-acylethanolamines (PEA and OEA) levels were significantly higher in obese, insulin-resistant subjects as compared to normal weight controls (Figure 1). Similar changes were also found when salivary endocannabinoid and *N*-acylethanolamine measurements were expressed as pmol/mg of total salivary lipids (data not shown). Conversely, in the same subjects, only fasting AEA and OEA plasma levels were higher in obese than in controls (Figure 1). Interestingly, compared to normal weight subjects, the increase was significantly higher in obese salivary than plasma samples for all the compounds analyzed but OEA (Figure 1).

There were no significant correlations between fasting salivary 2-AG and *N*-acylethanolamines and their respective plasma levels. However, a trend towards a significant correlation was found between salivary and plasma AEA levels (r = 0.39, *p* = 0.068).

**Figure 2:**
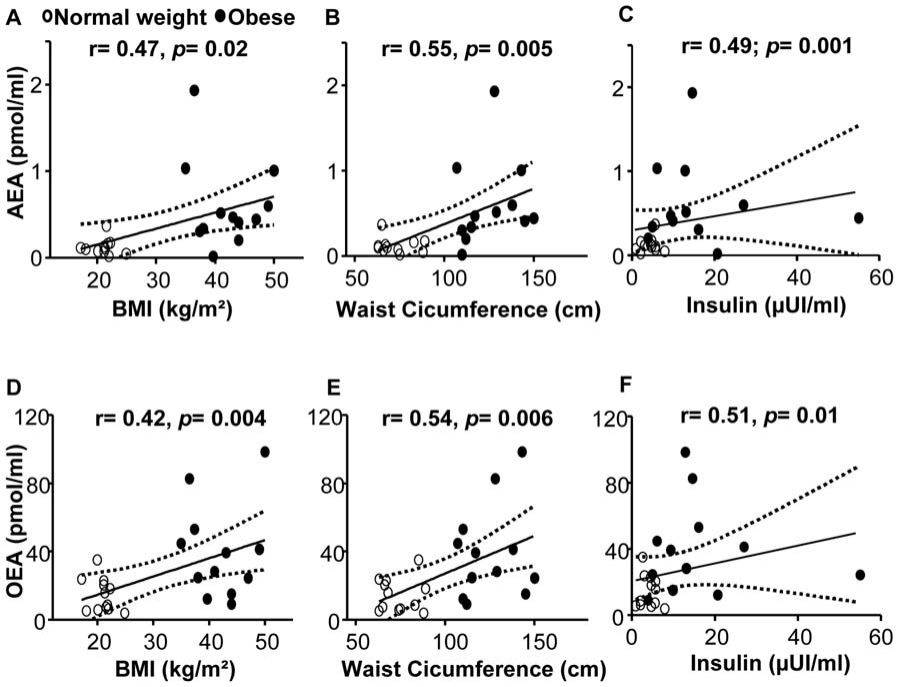
Relationship of salivary AEA and OEA levels with anthropometric and metabolic parameters. Fasting salivary AEA and OEA correlation with body mass index (BMI) (**A, D**), waist circumference (**B, E**) and fasting insulin levels (**C, F**).

Similarly to what has been previously reported for plasma measurements [7], [9], [10], fasting salivary AEA and OEA levels were significantly correlated with BMI, waist circumference, and fasting insulin (Figure 2). Fasting salivary 2-AG levels positively correlated with waist circumference (r = 0.50, *p* = 0.002) and insulin (r = 0.48, *p* = 0.027), while fasting salivary PEA did not correlate with any of these variables (data not shown).

### The ECS is present in human salivary glands

**Figure 3:**
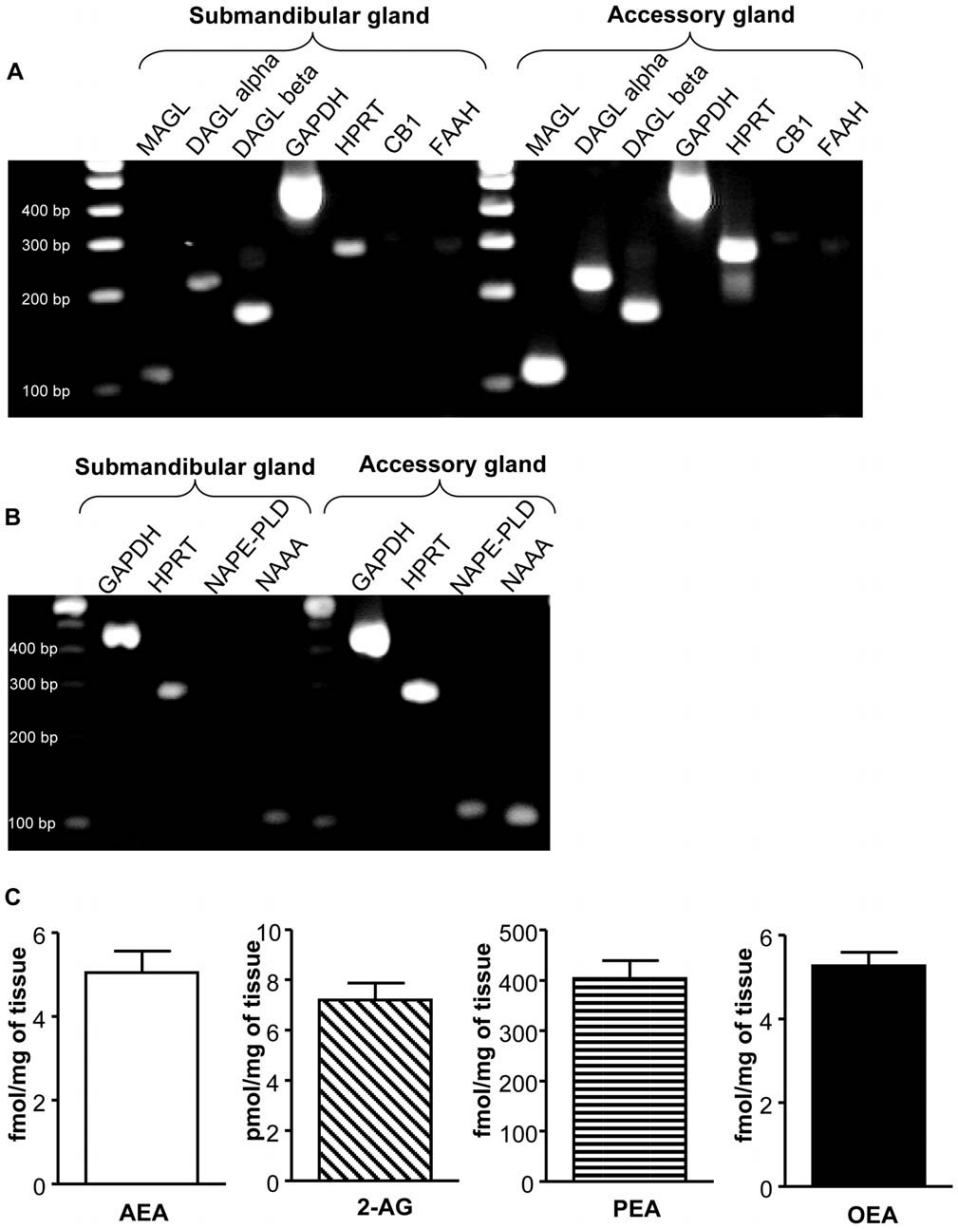
The ECS is present in human salivary glands. (**A**) RT-PCR for CB_1_, FAAH, MAGL, DAGL alpha, DAGL beta and housekeeping genes GAPDH and HRPT performed on submandibular and accessory salivary glands. (**B**) RT-PCR for NAPE-PLD and NAAA performed on submandibular and accessory salivary glands. A 100-bp ladder is shown. (**C**) Endocannabinoids and related *N*-acylethanolamines content in submandibular salivary glands. CB_1_: Cannabinoid receptor type 1, DAGL alpha: Diacylglycerol lipase alpha, DAGL beta: Diacylglycerol lipase beta, FAAH: Fatty acid amide hydrolase, GAPDH: glyceraldehyde-phosphate dehydrogenase, HPRT: hypoxanthine phosphoribosyl transferase, MAGL: Monoacylglycerol lipase, NAAA: N-acylethanolamine-hydrolyzing acid amidase, NAPE-PLD: N-acyl phosphatidylethanolamine phospholipase D.

Gene expression analysis of samples from human submandibular and salivary accessory glands revealed that MAGL, DAGL alpha and beta, FAAH and CB_1_ mRNAs were all expressed in saliva-producing organs (Figure 3A). Similarly, gene expression of enzymes involved in the synthesis of AEA, OEA and PEA, such as NAPE-PLD [28], and in their degradation, such as NAAA [29], were also present in salivary glands (Figure 3B). However, while MAGL, DAGL alpha and beta mRNAs were highly expressed, particularly in the accessory glands, mRNA levels of expression of CB_1_, FAAH, NAPE-PLD and NAAA were relatively low. AEA, 2-AG, PEA and OEA were also detectable in the gland tissues (Figure 3C).

### Salivary endocannabinoids and N-acylethanolamines meal-related responses

**Figure 4:**
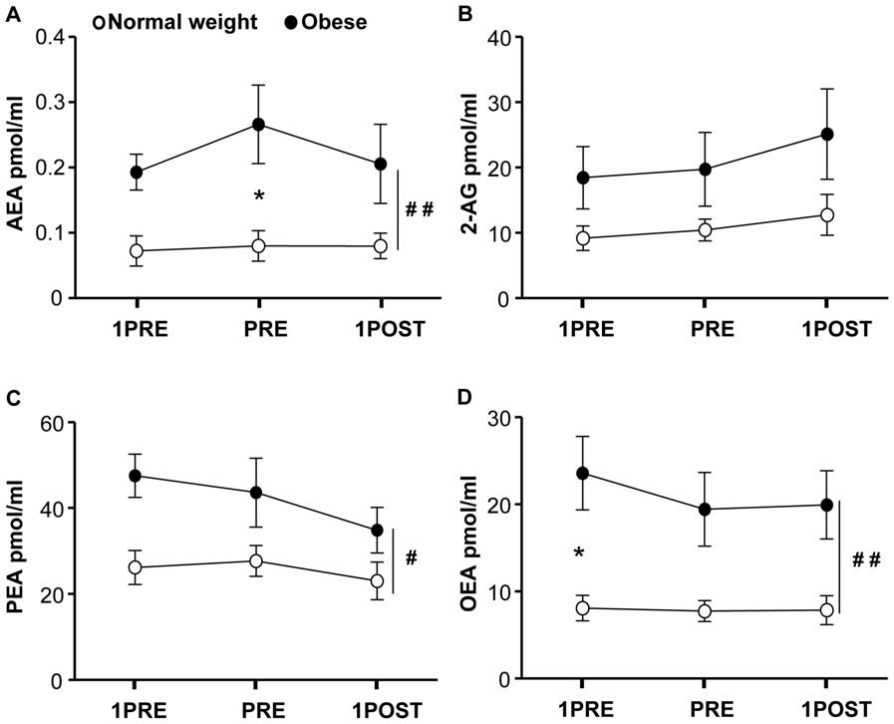
Changes in salivary endocannabinoids and N-acylethanolamines levels in response to a meal. Salivary AEA (**A**), 2-AG (**B**), OEA (**C**) and PEA (**D**) levels 1 h before (1 h PRE), immediately before (PRE) and 1 h after the termination of the meal (1 h POST). *p<0.05 normal weight *vs.* obese at a specific time point; ^#^ *p*<0.05, ^##^ *p*<0.01 normal weight *vs.* obese.

Normal weight and obese subjects had similar energy intake (normal weight: 2851.21±37.68 kJ *vs* obese: 2876±87.92 kJ, *p* = 0.8). Whatever the time point analyzed, salivary AEA, PEA and OEA levels were significantly higher in obese than in normal weight subject (Figure 4). However, salivary endocannabinoids and *N*-acylethanolamines levels did not change in response to the meal, neither in normal weight nor in obese (Figure 4).

### Salivary endocannabinoids and N-acylethanolamines weight loss-related responses

**Figure 5:**
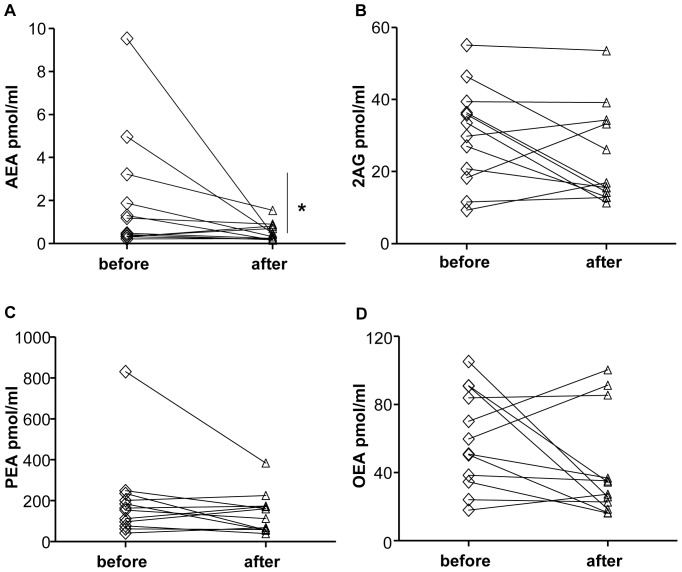
Changes in salivary endocannabinoids and N-acylethanolamines levels in response to body weight loss. Salivary AEA (**A**), 2-AG (**B**), PEA (**C**) and OEA (**D**) before and after body weight loss obtained through a 12 weeks life-style intervention program. *p<0.05 before *vs.* after the lifestyle intervention program.

The characteristics of the 12 obese subjects before and after the 12-weeks lifestyle intervention program are shown in Table 1. The compliance to the program led to significantly reduced body weight, BMI, waist circumference as well as systolic blood pressure (Table 1). It also significantly ameliorated fasting blood glucose, triglycerides, aspartate aminotransferase, alanine aminotransferase and gamma-glutamyl transferase (Table 1). Notably, at the end of the 12-weeks program the subjects had a significant reduction of fasting salivary AEA levels (*p*<0.05, Figure 5A), while salivary 2-AG, OEA and PEA did not significantly change (Figure 5B–D). Studies carried out in animal models suggest that AEA and OEA might have opposing roles in energy balance regulation [1], [2], [30], [31], thus the relationship between the ratio of salivary OEA/AEA levels and the percentage of body weight loss was also analyzed, but only a non-significant trend for a positive correlation was found (r: 0.44, *p* = 0.151).

## Discussion

Saliva collection is often used as a rapid, non-invasive diagnostic tool for the analysis of biomarkers in the clinical practice, and salivary samples can provide a cost-effective approach for the screening of large populations [11], [12].

Here we demonstrate that endocannabinoids and related *N*-acylethanolamines can be reliably detected and quantified in human saliva. Similarly to what already reported for circulating levels in the blood [7], [9], [10], the salivary concentration of AEA and OEA were significantly increased in obese, insulin-resistant subjects as compared to normal weight controls. Fasting salivary, but not plasma levels of 2-AG and PEA were also higher in obese patients than controls. Interestingly, obese subjects had actually higher levels of endocannabinoids and related *N*-acylethanolamines in saliva than plasma. The parallel increase in salivary AEA, PEA and OEA may actually suggest reduced FAAH activity in saliva or buccal cavity of obese subjects as a possible underlying mechanism, although this has not been tested in the present study. In addition, fasting salivary AEA and OEA levels directly correlated with BMI, waist circumference and fasting insulinemia, while a strong tendency towards a direct correlation between fasting salivary and plasma AEA levels was found.

Differently from our previous observations concerning the relationship between plasma AEA and food intake in humans [10], we were unable to observe any significant change in salivary AEA, 2-AG or *N*-acylethanolamines in response to the consumption of a meal, suggesting that this pool of endocannabinoids and related compounds is not affected by the feeding status of the subject. However, the relatively constant levels of salivary endocannabinoids observed during the meal study suggest that saliva might represent a good source of biological material for diagnostic purposes involving assessment of the ECS. Indeed, besides the easier access to saliva than plasma, the use of salivary samples would not require a tight control of the feeding status of the patients, which might represent an important source of variability in plasma measurements [10]. These findings also imply that plasma and salivary endocannabinoids might originate from separate compartments and serve different roles. In support of an autonomous production of endocannabinoids and related *N*-acylethanolamines in saliva, we have also found that the ECS, with its ligands, receptors and enzymes, localizes within the human salivary glands. However, further studies will have to detail the exact localization of the different components of the system within these tissues.

Interestingly, it has been recently shown that the administration of AEA or 2-AG in mice increases gustatory nerve responses to sweeteners in a CB_1_-dependent manner without affecting responses to salty, sour, bitter, and umami compounds [32]. In the same study, CB_1_ receptors were localized on taste bud cells expressing sweet taste receptors [32]. Thus, taking this evidence into account, it is possible that salivary endocannabinoids might work as sweet taste enhancers in humans as well. Another possibility that is worth mentioning is that the composition of the meal and in particular its fat content might affect salivary endocannabinoid and *N*-acylethanolamines levels, since dietary fat can influence the endocannabinoid and *N*-acylethanolamines pool in different tissues [24], [33], [34]. Finally, the ECS is also known to affect salivary secretion [20], [22], [35]. Interestingly, salivary secretion differs between normal weight and obese subjects [36]–[39], implying that the ECS might have a role in determining such differences.

Importantly, a mean body weight loss of 5% obtained at the end of a 12 weeks lifestyle intervention program, significantly decreased salivary AEA levels, without affecting any of the other compounds measured.

These are the first findings associating a decrease in salivary AEA with body weight loss, while the data provided so far on the response of plasma endocannabinoids to body weight loss induced by lifestyle, dietary programs have been inconsistent. For instance, Engeli and colleagues showed that in obese women fasting circulating endocannabinoid levels were not affected by a 5% weight loss obtained after 13–15 weeks of dietary weight reduction protocol [40]. The study was however carried out only once the subjects had maintained their weight stable for 3 months [40]. In contrast, in viscerally obese men, 1 year lifestyle intervention led to 7% weight loss and to a significant decrease in plasma AEA and 2-AG levels [41]. Probably such inconsistency across the studies published so far depends on several factors, including the rate of body weight loss, the point in time when the endocannabinoids measurement was carried out respect to when the body weight loss goal was reached and the gender of the studied subjects. Interestingly, salivary OEA changes were variable among the subjects and did not mirror AEA changes in response to body weight loss. A condition that might affect OEA levels is sleep apnea, since subjects suffering from this disorder have increased plasma OEA [42]. Among the subjects recruited for the lifestyle intervention study, 5 suffered from sleep apnea. However, their salivary OEA levels were comparable to subjects without the disorder (data not shown), suggesting that this might not be the reason why salivary OEA behaves differently from salivary AEA. On the other hand, several animal studies have suggested that AEA and OEA might have opposing roles in the regulation of energy balance and metabolism [1], [2], [30], [31]. While, as previously mentioned, studies assessing AEA and OEA levels in humans are, although sparse, generally showing concordant changes between these two compounds in plasma [7], [9], [43]. Here, we observed that the salivary OEA/AEA ratio tended to positively correlate with the amount of body weight loss. This observation, although potentially interesting, is not conclusive and a greater number of subjects will be required to verify whether such ratio might be a marker of body weight loss.

Finally, because of the low number of subjects constituting the studied cohorts, we were unable to verify whether gender differences exist in the levels of salivary endocannabinoids and related *N*-acylethanolamines as recently reported for these measurements in plasma [44].

Nevertheless, the present findings overall indicate that salivary AEA might be a useful biomarker in human obesity, in particular considering that salivary samples are easy to collect, require a non-invasive procedure advantageous when performing studies in obese subjects in whom venipuncture may be difficult, and can be repeatedly collected at home by the patient during a therapeutic intervention. This type of tool could therefore be used to better phenotype the obese population, assess responses to treatment, or to further study the physiology of the ECS in humans, by investigating salivary endocannabinoid responses under various conditions.
